# Association between Highly Active Antiretroviral Therapy and Type of Infectious Respiratory Disease and All-Cause In-Hospital Mortality in Patients with HIV/AIDS: A Case Series

**DOI:** 10.1371/journal.pone.0138115

**Published:** 2015-09-17

**Authors:** Renata Báez-Saldaña, Adriana Villafuerte-García, Pablo Cruz-Hervert, Guadalupe Delgado-Sánchez, Leticia Ferreyra-Reyes, Elizabeth Ferreira-Guerrero, Norma Mongua-Rodríguez, Rogelio Montero-Campos, Ada Melchor-Romero, Lourdes García-García

**Affiliations:** 1 Centro de Investigación sobre Enfermedades Infecciosas, Instituto Nacional de Salud Pública, Cuernavaca, Morelos, México; 2 Servicio Clínico de Neumología Oncológica, Instituto Nacional de Enfermedades Respiratorias, México, Distrito Federal, México; 3 División de Posgrado, Facultad de Medicina, Universidad Nacional Autónoma de México, México, Distrito Federal, México; 4 División de Posgrado, Facultad de Odontología, Universidad Nacional Autónoma de México, México, Distrito Federal, México; Azienda ospedaliero-universitaria di Perugia, ITALY

## Abstract

**Background:**

Respiratory manifestations of HIV disease differ globally due to differences in current availability of effective highly active antiretroviral therapy (HAART) programs and epidemiology of infectious diseases.

**Objective:**

To describe the association between HAART and discharge diagnosis and all-cause in-hospital mortality among hospitalized patients with infectious respiratory disease and HIV/AIDS.

**Material and Methods:**

We retrospectively reviewed the records of patients hospitalized at a specialty hospital for respiratory diseases in Mexico City between January 1st, 2010 and December 31st, 2011. We included patients whose discharge diagnosis included HIV or AIDS and at least one infectious respiratory diagnosis. The information source was the clinical chart. We analyzed the association between HAART for 180 days or more and type of respiratory disease using polytomous logistic regression and all-cause hospital mortality by multiple logistic regressions.

**Results:**

We studied 308 patients, of whom 206 (66.9%) had been diagnosed with HIV infection before admission to the hospital. The CD4+ lymphocyte median count was 68 cells/mm^3^ [interquartile range (IQR): 30–150]. Seventy-five (24.4%) cases had received HAART for more than 180 days. *Pneumocystis jirovecii* pneumonia (PJP) (n = 142), tuberculosis (n = 63), and bacterial community-acquired pneumonia (n = 60) were the most frequent discharge diagnoses. Receiving HAART for more than 180 days was associated with a lower probability of PJP [Adjusted odd ratio (aOR): 0.245, 95% Confidence Interval (CI): 0.08–0.8, p = 0.02], adjusted for sociodemographic and clinical covariates. HAART was independently associated with reduced odds (aOR 0.214, 95% CI 0.06–0.75) of all-cause in-hospital mortality, adjusting for HIV diagnosis previous to hospitalization, age, access to social security, low socioeconomic level, CD4 cell count, viral load, and discharge diagnoses.

**Conclusions:**

HAART for 180 days or more was associated with 79% decrease in all-cause in-hospital mortality and lower frequency of PJP as discharge diagnosis. The prevalence of poorly controlled HIV was high, regardless of whether HIV was diagnosed before or during admission. HIV diagnosis and treatment resources should be improved, and strengthening of HAART program needs to be promoted.

## Introduction

Globally, highly active antiretroviral therapy (HAART) has allowed for improved control of human immunodeficiency virus (HIV) infection and increased survival of infected individuals, as it extends virological suppression, promotes immune reconstitution and decreases the incidence of opportunistic infections, hospitalization, and mortality [1–3]. Therefore, a global initiative to scale up priority HIV/AIDS interventions in the health sector was launched in 2006, by which the member states agreed to work towards the goal of “universal access to comprehensive prevention programs, treatment, care and support” [4].

Concurrent to the benefits of HAART, there is evidence that frequency of HIV-related respiratory diseases as cause of hospitalization has changed [5]. In high resource settings, chronic obstructive respiratory disease (COPD), lung cancer, and immune reconstitution syndrome and its consequences are presently more frequent. In contrast, in regions where access to HAART is limited, opportunistic infections continue to rank first [6, 7].

In Mexico, with an estimated HIV prevalence among adults of 0.24% for 2012, all patients with AIDS or CD4+ cells below 350 cell/mm3 receive HAART paid by the government since 2003, and as of 2012 coverage was reported to be 85% [8]. However, access to HAART is delayed attributed to late HIV diagnosis due to late testing and to late presentation to care after diagnosis [9].

The association between HAART and respiratory disease has not been systematically evaluated in low and medium resource settings. Therefore, the objective of this study was to describe the association between HAART and the type and frequency of respiratory diseases on hospital discharge and all-cause in-hospital mortality among patients with infectious respiratory disease and HIV/AIDS.

## Materials and Methods

### Design, study setting, and population

The National Institute of Respiratory Diseases is a referral tertiary-care hospital for patients with respiratory diseases that provides ambulatory and hospital medical care to population with and without social security, mostly living in Mexico City and surrounding states. We conducted a retrospective review of consecutive hospitalized patients admitted between January 1st, 2010 and December 31st, 2011 whose discharge diagnosis included HIV /AIDS and at least one respiratory disease.

The information source was the clinical chart. We excluded incomplete clinical charts as defined by the Official Mexican Standards for clinical charts (NOM-168-SSA1-1998) [10]. Collection of information was conducted through review of patient clinical files by using a chart-abstraction tool. Chart review was conducted by trained personnel. Incomplete and inaccurate clinical charts were queried and corrected by one of the authors (AVG) through checking clinical and laboratory records. AVG reviewed laboratory records to identify diagnoses that had not been recorded in the clinical file and that might have been reported after the patient´s discharge. Data obtained was double checked to ensure quality, completeness, and validity by two of the authors (AVG and RBS). There were no transfers, departures against medical advice or discontinuation of care. When a patient had previous hospitalizations, only information from the last hospitalization was considered.

### Criteria for diagnosis

Diagnosis of respiratory diseases was based on standardized diagnosis algorithms considering clinical manifestations, imaging findings (chest radiography and/or chest computed tomography), microbiological, cytological, and histological results, as well as regional epidemiology. Diagnosis of *Pneumocystis jirovecii* pneumonia (PJP) was relied on microscopic visualization of the characteristics cysts or trophic forms on stained bronchoalveolar lavage (BAL) or biopsy specimens. Cytomegalovirus (CMV) pneumonia, took into account pulmonary interstitial infiltrates, identification of multiple CMV inclusion bodies in lung tissue biopsy obtained by bronchoscopy or thoracoscopy, and the absence of other pathogens more commonly associated with pneumonitis in HIV population. Community-acquired pneumonia was considered in patients who presented with new pulmonary opacities in a chest radiograph associated with at least one of the following: new or increased cough, fever or hypothermia, leukocytosis or leukopenia, and no history of hospitalization during the two weeks prior to admission. Pathogen isolation and identification in expectorated sputum, BAL, blood culture or pleural effusion samples was conducted on the basis of clinical and epidemiologic clues. Tuberculosis was diagnosed based on the identification of acid-fast bacillus (AFB) and/or positive cultures for *Mycobacterium tuberculosis* in expectorated sputum or BAL samples, or by microorganism histopathological identification. In paucibacillary or extrapulmonary cases, molecular biology samples (Gene Xpert) based on DNA amplification for *Mycobacterium tuberculosis* identification was used. The diagnosis of pulmonary histoplasmosis was established by direct examination of BAL samples or lung biopsy specimens, documenting the presence of a histiocyte-rich inflammatory cell infiltrate with numerous fungi morphologically (yeasts) consistent with histoplasmosis and confirmed by cultures and microscopic examination positive for *Histoplasma capsulatum*. Pulmonary aspergillosis was relied on the presence of necrotizing pneumonitis and numerous septate fungal hyphae with *Aspergillus fumigatus* isolated from a fungal culture of lung tissue. The diagnosis of pulmonary cryptococcosis was established by initial blood cultures growing yeast within 48 hours of admission, identified as *Cyrptococcus neoformans* which was also isolated from the BAL samples and/or biopsy specimens from transbronchial biopsy. All lung tissue sections were stained with haematoxylin-eosin (HE) and histochemically stained with periodic acid-Schiff, mucus card Red, and Grocott’s methenamine silver. Diagnosis of neoplasms, such as Kaposi’s sarcoma, non-Hodgkin’s lymphoma, and others, was based on the histopathology and confirmed immunohistochemistry of bronchial, transbronchial or open-lung biopsy specimens [11–13].

### HIV testing

Following WHO recommendation on provider-initiated HIV testing and counselling for concentrated HIV epidemics (HIV prevalence consistently over 5% in at least one defined subpopulation but below 1% in pregnant women in urban areas), HIV testing was offered to patients who referred HIV risk factors, and to patients whose clinical presentation might result from underlying HIV infection [14]. The patients were notified orally that testing was planned, advised of the indication for testing and the implications of positive and negative test results, and offered an opportunity to ask questions and to decline testing. Individuals must specifically have declined the HIV test if they did not want it to be performed. An initial enzyme immunossay was used to detect HIV antibodies; all positive tests were confirmed by enzyme immunoassay and western blot [15].

### Exposure variables

#### Definition of HAART

HART duration of therapy was ascertained from self-report as described in the clinical chart. Treatment with HAART for 180 days or more was defined when a patient had received the combination of at least three antiretroviral drugs pertaining to at least two different classes for 180 days or more when admitted to the hospital. This cutoff point was selected because it provides enough time to observe the effects of antiretroviral treatment on the symptoms of the disease [16].

In the case of HIV infected patients who presented with respiratory infection and received a simultaneous HIV infection diagnosis, HAART was initiated following national guidelines. In most cases, treatment was started within the first 14 days of antibiotic treatment. In the case of patients already receiving HAART, the continuation schedule was individualized, although in general, its administration continued along with specific treatment for the respiratory disease. In all of the cases, the treatment schedule was administered according to national and international standards [17, 18].

#### Covariates

We extracted the following information from the clinical charts: sociodemographic (gender, age, age ≥50 years old, low socioeconomic level, illiteracy and elementary school, and access to social security), epidemiologic (current smoking; current illegal drug use; HIV related risk behavior, and clinical (HIV diagnosis previous to admission; time elapsed between HIV diagnosis and hospital admission [days]; CD4+ cell counts [cells/mm^3^]; CD4+ cell count < 200/mm^3^; viral load [<50 copies/ml or undetectable viral load]; time to respiratory disease [time elapsed between the onset of symptoms and hospital admission, days]; and hospital stay [days]). Socioeconomic level was based on evaluation of family income, occupation, educational level, number of family members living at the current residence, number of school-age children in the family, construction material of current residence, ownership of current residence, home location (urban or rural), and service availability (electricity, paved roads, piped-in water, and sewage). This information was investigated by a social worker on patient admission and graded on a 7-point scale. We defined individuals from low socioeconomic level as those who were graded points one to three. Current illegal drug use referred to smoking marijuana and inhaled cocaine. There were no patients referring intravenous drug usage. The only HIV related risk behavior was men having sex with men. Given the retrospective design of the study, we did not have information on treatment adherence. Therefore, we used fully controlled viral replication as indicator of full adherence. Fully controlled viral replication was considered when viral load was <50 copies/ml or undetectable viral load. Time to respiratory disease was defined as the time (days) elapsed between the onset of the symptoms and diagnosis of respiratory disease.

### Outcomes

Discharge diagnoses were classified in six groups: 1) PJP; 2) Bacterial community-acquired pneumonia; 3) Tuberculosis (pulmonary and disseminated); 4) Other infectious respiratory diseases caused by a single pathogen; 5) Non-infectious respiratory diseases; and 6) Mixed (combination of one or more of previous diagnoses in a single patient). Mortality refers to all-cause in-hospital mortality. Respiratory diagnoses on discharge and causes of death were coded according to the International Classification of Diseases Tenth Revision (ICD-10) [19].

### Data Analysis

We compared characteristics of patients for whom we obtained a complete clinical chart with those on whom the chart was incomplete or unavailable. We classified discharge diagnoses according to six groups (PJP; bacterial community-acquired pneumonia; tuberculosis (pulmonary and disseminated); other infectious respiratory diseases, non-infectious respiratory diseases, and mixed). Given that the median age of our study population was 34 years (Interquartile range [IQR] 29–42) and therefore not at risk for non-infectious respiratory diseases, we excluded this group from bivariate and multivariate analyses. We conducted bivariate analyses to examine differences between respiratory diagnoses (PJP; bacterial community-acquired pneumonia; tuberculosis; other infectious respiratory diseases; and mixed); patients with HIV diagnosis previous to admission as compared to patients who were diagnosed during hospital stay; and all-cause in-hospital fatalities as compared to surviving patients. Patient characteristics were compared according to each study group using chi-squared tests for categorical variables and Kruskal-Wallis test for continuous variables with non-normal distribution. To investigate the relation between HAART for more than 180 days on respiratory diagnosis, we used polytomous logistic regression with a five-level outcome variable (PJP, tuberculosis, bacterial community-acquired pneumonia, other infectious pneumonia [reference category], and mixed). We calculated adjusted odds ratios (aORs) and 95% confidence intervals (95% CIs). The saturated polytomous regression model included the following variables: age >50 years old, low socioeconomic level, access to social security, current smoker, current illegal drug user, diagnosis of HIV infection previous to hospitalization, HAART for 180 days or more, < 50 viral copies/ml or undetectable viral load, hospital stay (days), current smoking and time of respiratory disease (days).We used the Akaike Information Criterion (AIC) for the final model selection. The model excluded access to social security, hospital stay and time of the respiratory disease. Effect modification and interaction were evaluated but no significant effects were identified using the likelihood ratio test, comparing models with and without multiplicative interaction terms.

For all the study population, for patients who had been diagnosed with HIV infection previous to admission, and for patients with bacterial community acquired pneumonia association between HAART for more than 180 days and all-cause in-hospital mortality was investigated by multivariate unconditional logistic regression. We analysed patients who had been diagnosed with HIV infection prior to admission separately to try to separate the effect that knowledge of patients´ HIV status might have on more invasive and informative diagnostic approach or better management for their opportunistic infection. Discharge diagnosis was stratified as a five-level variable (bacterial community-acquired pneumonia; other infectious diseases; PJ pneumonia; mixed; and tuberculosis [reference category]. The logistic regression models were validated by evaluating their suitability, model specificity and multicollinearity. Variables included in the models were those with *p*-values ≤0.20 in the bivariate analysis or with biological plausibility, and covariates were achieved using a hierarchical backward elimination approach. The variable association was expressed as aOR at 95% CI.

With 54 all-cause in-hospital fatalities and 254 surviving patients, the study had 93.4% power at a 5% significance level to test the hypothesis that the proportion of patients receiving HAART for more than 180 days among surviving patients (28.0%) was different from that among all-cause in-hospital fatalities (7.4%).

All analyses were performed using the STATA 13.1 statistical software package (StataCorp LP, College Station, TX, USA).

### Ethical considerations

This study was approved by the Research Ethical Committee of the National Institute for Respiratory Diseases (Instituto Nacional de Enfermedades Respiratorias) (approval number C53-11).

## Results

Over the duration of the study, 351 patients with HIV infection or AIDS and at least one respiratory pathology were discharged from the hospital, of whom 91.7% (n = 322) had complete clinical charts. Patients with incomplete clinical charts (8.3% [29/351]) were not different in terms of age and gender compared with those cases with complete clinical charts.

Of the 322 patients, 44.1% (n = 142) were diagnosed with PJP; 19.6% (n = 63) with tuberculosis, 18.6% (n = 60) with bacterial community acquired pneumonia; 7.1% (n = 23) with mixed diagnoses (all combinations including at least one infectious agent); 6.2% (n = 20) with other infectious respiratory disease different from PJP, tuberculosis or community acquired pneumonia and caused by a single pathogen; and 4.4% (n = 14) with non-infectious diseases. We were able to isolate a pathogen in 40% (24/60) of patients with bacterial community acquired pneumonia: *Streptococcus pneumoniae* (n = 9); *Pseudomonas aeruginosa* (n = 7); *Staphylococcus aureus* (n = 5); and *Klebsiella pneumoniae* (n = 3). Discharge diagnoses are detailed in S1 Table.

Given that the median age of the 322 patients was 34 years (IQR 29–42) and therefore not at risk for non-infectious respiratory diseases, we excluded the group of non-infectious diseases from further analyses. Therefore our study population is comprised of 308 patients whose discharge diagnoses included at least one infectious disease.

Table 1 shows sociodemographic and clinical characteristics of all patients according to respiratory diagnosis. Of the 308 patients, the median (interquartile range) age was 34 (29–42) years, 88.0% were male, most (80.2%) came from low socioeconomic level, and 66.9% had been diagnosed with HIV infection previous to hospitalization. Sixteen percent of patients referred current illegal drug use although non referred intravenous drug use. The median (IQR) for CD4+ lymphocytes was 68 (30–150) cells/mm^3^, 81.8% with CD4 counts below 200 cells/mm^3^, and 18.1% had less than 50 viral copies/ml or undetectable viral load. Twenty-four percent of cases had received HAART for more than 180 days; of these, 26.7% (n = 20) had fully controlled viral replication. Twelve patients referred having received influenza vaccine; two patients pneumococcal vaccine, ten patients PJP prophylaxis, and fourteen patients tuberculosis prophylaxis. Seventeen percent of patients died during hospitalization. Comparison of patient characteristics according to respiratory diagnoses revealed differences for socioeconomic level (patients with tuberculosis with the higher proportion of low socioeconomic level); current illegal drug usage (patients with mixed etiologies referring highest proportion of usage); diagnosis of HIV infection previous to hospitalization (patients with tuberculosis with highest proportion of prior diagnosis); HAART for 180 days or more (patients with other infectious diseases with the highest proportion); viral loads below 50 copies/ml or undetectable loads (patients with other infectious diseases with the highest proportion of low viral loads); and death during hospitalization (patients with bacterial community acquired pneumonia with the highest proportion of mortality).

**Table 1:** Characteristics of HIV/AIDS patients in a reference hospital for respiratory diseases in Mexico City, from January 2010 to December 2010, according to discharge respiratory diagnoses.

Characteristics	Total	PJ pneumonia	Bacterial community-acquired pneumonia	Tuberculosis	Other infectious diseases	Mixed	p value^b^
	n/tot (%)	n/tot (%)	n/tot (%)	n/tot (%)	n/tot (%)	n/tot (%)	
Men	271/308 (88)	120/142 (84.5)	56/60 (93.3)	56/63 (88.9)	19/20 (95)	20/23 (87)	0.373
Age, years (median (IQR)	34 (29–42)	33.5(28–41)	35 (32–42.5)	34 (27–43)	35 (30.5–39.5)	35 (30–46)	0.421^c^
Age ≥50 years old	35/308 (11.4)	13/142 (9.2)	8/60 (13.3)	8/63 (12.7)	2/20 (10)	4/23 (17.4)	0.755
Low socioeconomic level	247/308 (80.2)	108/142 (76.1)	48/60 (80)	60/63 (95.2)	15/20 (75)	16/23 (69.6)	0.014
Access to Social Security	183/308 (59.4)	94/142 (66.2)	28/60 (46.7)	37/63 (58.7)	13/20 (65)	11/23 (47.8)	0.081
Illiterate and elementary school	85/308 (27.6)	32/142 (22.5)	20/60 (33.3)	22/63 (34.9)	6/20 (30)	5/23 (21.7)	0.292
Men having sex with men	47/109 (43.1)	19/51 (37.2)	9/18 (50.0)	11/26 (42.3)	3/6 (50.0)	5/8 (62.5)	0.661
Current smoker	113/308 (36.7)	46/142 (32.4)	26/60 (43.3)	23/63 (36.5)	5/20 (25)	13/23 (56.5)	0.119
Current illegal drug user (marijuana and/or cocaine)	49/308 (15.9)	19/142 (13.4)	5/60 (8.3)	11/63 (17.5)	6/20 (30)	8/23 (34.8)	0.014
Diagnosis of HIV infection previous to hospitalization	206/308(66.9)	79/142 (55.6)	40/60 (66.7)	55/63 (87.3)	17/20 (85)	15/23 (65.2)	<0.001
Time elapsed between HIV diagnosis and hospital admission, days [median (IQR)]	365(60.0–219)	365 (38–2190)	365 (90–2190)	365(150–109)	365 (150–1095)	60 (20–365)	0.344 ^c^
HAART for 180 days or more	75/308 (24.4)	23/142 (16.2)	16/60 (26.7)	23/63 (36.5)	10/20 (50)	3/23 (13)	0.001
CD4+ cells/mm^3^ [median (IQR)]	68 (30–150)	69 (30–149)	56 (23–187)	71 (34–139)	70 (34–302)	54 (20–98)	0.739 ^c^
< 200 CD4+ cells/mm^3^	247/302 (81.8)	119/140 (85)	44/58 (75.9)	49/62 (79)	14/19 (73.7)	21/23 (91.3)	0.299
Viral load <50 copies/ml or undetectable	54/298 (18.1)	24/137 (17.5)	8/57 (14)	11/62 (17.7)	9/19 (47.4)	2/23 (8.7)	0.011
Time to respiratory disease (days) [median (IQR)] ^a^	30(15–60)	21(15–60)	21(15–58.5)	32(20–90)	11(3–60)	45(8–60)	0.074 ^c^
Hospital stay (days) [median (IQR)]	14(9–24)	13(10–23)	14(9–27)	14(9–21)	13.5(4.5–24.5)	21(16–35)	0.067 ^c^
Death during hospitalization	54/308 (17.5)	17/54 (12.0)	27/54 (45.0)	4/54 (6.3)	3/54 (15.0)	3/54 (13.0)	<0.001

HIV, Human Immunodeficiency Virus; AIDS, Acquired Immunodeficiency Syndrome; IQR, interquartile range; PJ, *Pneumocystis jirovecii*;

^a^Defined as the time elapsed between the onset of the symptoms and diagnosis of respiratory disease.

^b^Chi square test;

^c^Kruskal- Wallis test.

Table 2 compares characteristics of patients who were diagnosed with HIV previous to hospital admission with those of patients who were HIV diagnosed during hospitalization. Patients who were diagnosed prior to hospital admission were more likely to be younger, from low socioeconomic level, to have social security, to have received HAART for 180 days or more, to have higher counts of CD4+ cells, to be diagnosed on discharge with TB and less likely to be men having sex with men, current smokers, or to be diagnosed on discharge with PJP.

**Table 2:** Characteristics of HIV/AIDS patients in a specialty hospital for respiratory diseases in Mexico City, from January 2010 to December 2010, according to diagnosis of HIV infection previous to hospitalization.

Characteristics	Total	Diagnosis of HIV infection previous to hospitalization	Diagnosis of HIV infection during to hospitalization	p value^b^
	n/tot (%)	n/tot (%)	n/tot (%)	
Men	271/308 (88.0)	186/206 (90.3)	85/102 (83.3)	0.077
Age, years [median (IQR)]	34(29–42)	33.5 (28–40)	37 (31–44)	0.005^c^
Age >50 years old	35/308 (11.4)	19/206 (9.2)	16/102 (15.7)	0.093
Low socioeconomic level	247/308 (80.2)	177/206 (85.9)	70/102 (68.6)	<0.001
Access to Social Security	183/308 (59.4)	132/206 (64.1)	51/102 (50.0)	0.018
Illiterate and elementary school	85/308 (27.6)	53/206 (25.7)	32/102 (31.4)	0.297
Men having sex with men	47/109 (43.1)	27/78 (34.6)	20/31 (64.5)	0.004
Current smoker	113/308 (36.7)	67/206 (32.5)	46/102 (45.1)	0.031
Current illegal drug user (marijuana and/or cocaine)	49/308 (15.9)	37/206 (18.0)	12/102 (11.8)	0.162
CD4+ cells/mm^3^ [median (IQR)]	68 (30–150)	75 (34–163)	50 (26–131)	0.017 ^c^
< 200 CD4+ cells/mm^3^	247/302 (81.8)	164/204 (80.4)	83/98 (84.7)	0.364
HAART for 180 days or more	75/308 (24.4)	75/207 (36.2)	0/102 (0.0)	<0.001
Viral load <50 copies/ml or undetectable viral load	54/298 (18.1)	37/203 (18.2)	17/95 (17.9)	0.945
Time to respiratory disease (days) [median (IQR)]^a^	30 (15–60)	29 (10–60)	30 (15–60)	0.129 ^c^
Hospital stay (days) [median (IQR)]	14 (9–24)	14 (9–24)	16.5 (11–24)	0.195 ^c^
Discharge diagnosis				
PJ pneumonia	142/308 (46.1)	79/206 (38.3)	63/102 (61.8)	<0.001
Bacterial community-acquired pneumonia	60/308 (19.5)	40/206 (19.4)	20/102 (19.6)	0.968
Tuberculosis	63/308 (20.5)	55/206 (26.7)	8/102 (7.8)	<0.001
Other infectious diseases	20/308 (6.5)	17/206 (8.3)	3/102 (2.9)	0.075
Mixed	23/308 (7.5)	15/206 (7.3)	8/102 (7.8)	0.860

HIV, Human Immunodeficiency Virus; AIDS, Acquired Immunodeficiency Syndrome; IQR, interquartile range; PJ, *Pneumocystis jirovecii*;

^a^Defined as the time elapsed between the onset of the symptoms and diagnosis of respiratory disease.

^b^Chi square test;

^c^Kruskal- Wallis test.

The patients who died during their hospitalization compared with those who clinically improved were older, less likely to have social security, to have been diagnosed with HIV prior to admission, and to have received HAART for 180 days or more. These patients were more likely to have CD4+ cells below 200/mm^3^ and viral load above 50 copies/ml. They had longer hospital stay, and were less likely to be diagnosed with PJP or tuberculosis, and more likely to be diagnosed with bacterial community-acquired pneumonia (Table 3).

**Table 3:** Characteristics of HIV/AIDS patients in a specialty hospital for respiratory diseases in Mexico City, from January 2010 to December 2011, according to hospital discharge.

	Total	All-cause in-hospital fatalities	Discharge with clinical improvement	p value^b^
Characteristics	n/tot (%)	n/tot (%)	n/tot (%)	
Men	271/308 (88.0)	46/54 (85.2)	225/254 (88.6)	0.486
Age, years [median (IQR)]	34 (29–42)	37 (31–45)	34 (28–41)	0.028^c^
Age >50 years old	35/308 (11.4)	10/54 (18.5)	25/254 (9.8)	0.068
Low socioeconomic level	247/308 (80.2)	40/54 (74.1)	207/254 (81.5)	0.214
Access to Social Security	183/308 (59.4)	15/54 (27.8)	168/254 (66.1)	<0.001
Illiterate and elementary school	85/308 (27.6)	18/54 (33.3)	67/254 (26.4)	0.299
Men having sex with men	47/109 (43.1)	10/20 (50.0)	37/89 (41.5)	0.492
Current smoker	113/308 (36.7)	19/54 (35.2)	94/254 (37.0)	0.801
Drug user (marijuana and/or cocaine)	49/308 (15.9)	4/54 (7.4)	45/254 (17.7)	0.060
Diagnosis of HIV infection previous to hospitalization	206/308 (66.9)	25/54 (46.3)	181/254 (71.3)	0.001
HAART for 180 days or more	75/308 (24.4)	4/51 (7.4)	71/254 (28.0)	<0.001
CD4+ cells/mm^3^, [median (IQR)]	68 (30–150)	34 (16–64)	73 (34–172)	<0.001 ^c^
< 200 CD4+ cells/mm^3^	247/302 (81.8)	49/52 (94.2)	198/250 (79.2)	0.011
Viral load < 50 copies/ml or undetectable viral load	54/298 (18.1)	4/50 (8.0)	50/248 (20.2)	0.042
Time of the respiratory disease (days)[median (IQR)]^a^	30 (15–60)	29 (15–60)	30 (15–60)	0.656 ^c^
Hospital stay (days) [median (IQR)]	14 (9–24)	19 (12–27)	14 (9–23)	0.010 ^c^
Discharge diagnosis				
PJ pneumonia	142/308 (46.1)	17/54 (31.5)	125/254 (49.2)	0.018
Bacterial community-acquired pneumonia	60/308 (19.5)	27/54 (50.0)	33/254 (13.0)	<0.001
Tuberculosis	63/308 (20.5)	4/54 (7.4)	59/254 (23.2)	0.009
Other infectious diseases	20/308 (6.5)	3/54 (5.6)	17/254 (6.7)	0.758
Mixed	23/308 (7.5)	3/54 (5.6)	20/254 (7.9)	0.556

HIV, Human Immunodeficiency Virus; AIDS, Acquired Immunodeficiency Syndrome; IQR, interquartile range; PJ, *Pneumocystis jirovecii*;

^a^Defined as the time elapsed between the onset of the symptoms and diagnosis of respiratory disease.

^b^Chi square test;

^c^Kruskal- Wallis test.

Using polytomous logistic regression, we demonstrated that HAART was independently associated with reduced odds of PJP (Adjusted odds ratio (aOR) 0.245, 95% confidence interval (CI): 0.08–0.80) and reduced odds of mixed infections (aOR 0.172, 95%CI: 0.03–0.91) as a discharge diagnosis, adjusting for diagnosis of HIV infection prior to admission, age, socioeconomic level, illegal drug use, CD4+ cells/mm^3^, and viral load (Table 4).

**Table 4:** Association between respiratory disease and HAART for 180 days or more, adjusted for clinical characteristics, in HIV/AIDS patients from a specialty hospital for respiratory diseases in Mexico City from January 2010 to December 2011.

Characteristics	PJ pneumonia		Bacterial community-acquired pneumonia		Tuberculosis		Other infectious diseases	Mixed (at least one infectious disease)	
	aOR^a^	P-value	aOR^a^	P-value	aOR^a^	P-value	Reference outcome	aOR^a^	P-value
	(95%CI)		(95%CI)		(95%CI)			(95%CI)	
HAART for 180 days or more	0.245	0.02	0.386	0.141	0.435	0.181		0.172	0.038
	(0.08–0.8)		(0.11–1.37)		(0.13–1.48)			(0.03–0.91)	
Diagnosis of HIV infection previous to hospitalization	0.410	0.249	0.627	0.569	1.494	0.638		0.684	0.667
	(0.09–1.86)		(0.13–3.12)		(0.28–7.95)			(0.12–3.86)	
Age >50 years old	0.686	0.665	1.123	0.898	1.347	0.742		2.024	0.483
	(0.12–3.78)		(0.19–6.6)		(0.23–7.94)			(0.28–14.51)	
Low socioeconomic level	2.178	0.221	2.590	0.168	9.977	0.006		1.427	0.639
	(0.63–7.57)		(0.67–10)		(1.94–51.34)			(0.32–6.29)	
Illegal drug user (marijuana and/or cocaine)	0.248	0.022	0.169	0.013	0.374	0.127		0.913	0.899
	(0.08–0.82)		(0.04–0.69)		(0.11–1.32)			(0.22–3.72)	
< 200 CD4+ cells/mm^3^	0.890	0.865	0.440	0.256	0.617	0.503		1.364	0.762
	(0.23–3.4)		(0.11–1.82)		(0.15–2.53)			(0.18–10.17)	
Viral load < 50 copies/ml or undetectable	0.219	0.014	0.117	0.002	0.159	0.006		0.122	0.031
	(0.07–0.74)		(0.03–0.46)		(0.04–0.59)			(0.02–0.83)	

HAART, highly active antiretroviral therapy;

^a^ aOR, adjusted odds ratio using multivariated polytomous regression; 95% CI,95% confidence interval.

Using logistic regression, we demonstrated that HAART was independently associated with reduced odds (aOR 0.214, 95% CI 0.06–0.75) of all-cause in-hospital mortality, adjusting for HIV diagnosis previous to hospitalization, age, access to social security, low socioeconomic level, CD4 cell count, viral load, and discharge diagnoses, (Model 1, Table 5). We also observed that HAART was independently associated with reduced odds (aOR 0.194, 95% CI 0.05–0.76) of all-cause in-hospital mortality, among patients who had been diagnosed with HIV infection previous to hospitalization adjusting for the same covariates, (Model 2, Table 5). In these models, we observed that bacterial community acquired pneumonia was independently associated with increased odds (aOR 15.55, 95% CI 4.04–59.89 and aOR 23.75 95% CI 3.81–147.71) of all-cause in-hospital mortality, among all patients and among those who had been diagnosed with HIV prior to admission, respectively. Finally, we did not find significant association between HAART and all-cause in-hospital mortality among patients whose discharge diagnosis was bacterial community acquired pneumonia (aOR 0.092 (95% CI (0.01–1.60) adjusting by the same covariates.

**Table 5:** Association between all-cause in-hospital mortality and selected variables in HIV/AIDS patients admitted into a specialty hospital for respiratory diseases in Mexico City from January 2010 to December 2011.

Variables	Model 1	Model 2
	Overall	Patients with diagnosis of HIV infection previous to hospitalization
	n = 297	n = 202
	aOR^a^	aOR^a^
	(95%CI)	(95%CI)
HAART for 180 days or more	0.214^b^	0.194 ^b^
	(0.06–0.75)	(0.05–0.76)
Diagnosis of HIV infection previous to hospitalization	0.648	____
	(0.29–1.47)	____
Age >50 years old	3.650 ^b^	5.877 ^b^
	(1.32–10.06)	(1.46–23.62)
Access to Social Security	0.181^c^	0.151^c^
	(0.08–0.41)	(0.05–0.45)
Low socioeconomic level	0.633	0.356
	(0.26–1.54)	(0.10–1.25)
< 200 CD4+ cells/mm^3^	8.966^b^	3.203
	(1.82–44.27)	(0.61–16.72)
Viral load < 50 copies/ml or undetectable viral load	0.508	0.780
	(0.13–1.96)	(0.13–4.59)
Discharge diagnosis		
Bacterial community-acquired pneumonia	15.55^c^	23.75^c^
	(4.04–59.89)	(3.81–147.71)
Other infectious diseases	5.62	6.17
	(0.90–35.08)	(0.59–63.93)
PJ pneumonia	1.77	2.03
	(0.48–6.50)	(0.31–13.0)
Mixed	1.02	4.58
	(0.17–5.84)	(0.53–39.05)
Tuberculosis	1	1
	Reference	Reference

HAART, highly active antiretroviral therapy;

^a^ aOR, adjusted odds ratio using multivariate logistic regression; 95% CI, 95% confidence interval;

^b^ p value < 0.05,

^c^ p value < 0.001.

## Discussion

In the present study, we evaluated the association between HAART for 180 days or more and type of infectious respiratory diseases and all-cause in-hospital mortality in a specialty hospital for respiratory diseases. We chose to use HAART and not HIV diagnosis prior to admission because previous studies have documented delayed access to HAART among HIV diagnosed patients [9]. We found that patients receiving HAART for 180 days or more had a lower probability of being diagnosed with PJP and of dying during hospitalization.

Aligned with previous studies performed in Latin America and Mexico, and in contrast to European countries, we found that the majority of studied patients were at an advanced stage of the disease with low CD4+ counts and high viral loads [9, 20]. Almost a third of patients had not been diagnosed with HIV on admission to the hospital and among those diagnosed only 36.2% had received HAART for more than 180 days.

In contrast with prior evidence showing that community-acquired bacterial pneumonia and COPD are the most frequent pathologies in high resource settings [21], PJP was the most frequent diagnosis as has been observed in low and medium resources regions where late initiation of HAART has also been noted [5, 22].

HAART and effective prophylaxis for PJP are interventions with high impact on morbidity and mortality reduction in HIV-infected adults and have demonstrated cost-effectiveness in resource-limited settings [23–26]. HAART has been associated to reduced frequency of PJP [22, 27]. Mexican government offers universal HAART and PJP prophylaxis to HIV infected individuals. Late initiation of HAART in our setting may have several explanations. The main one is that patients are not tested until an opportunistic infection becomes apparent, as happened in 33.1% of cases in our series. Secondly, infected patients may not receive adequate health care due to inadequate linkage between testing and health care facilities, lack of training of personnel involved or limited drug supplies. This is supported by the observation that although Mexican government provides HAART together with PJP prophylaxis, of 75 patients with HAART for more than 180 days, only ten referred receiving PJP prophylaxis and other measures such as pneumococcal vaccine and tuberculosis prophylaxis were infrequent [17]. Another factor was treatment compliance, which can be poor in some patients due to side effects [22]. Our study design limited our ability to measure treatment adherence. We used fully controlled viral replication as an indicator of compliance. Results support the notion that treatment compliance is low. Additionally, many of the patients who had started HAART were in advanced stages of the disease and, therefore, were severely immunocompromised, which can also limit treatment effectiveness. Despite this disadvantaged situation, our findings show that receiving HAART for more than 180 days was independently associated with reduced odds of PJP and mortality.

As shown by other authors [28], we observed higher odds of all-cause in-hospital mortality associated to bacterial community-acquired pneumonia. Prior to HAART, bacterial community-acquired pneumonia was significantly more frequent in HIV patients versus those that were not infected. However, it has been documented that the risk for community-acquired bacterial pneumonia is clearly associated with a decrease in CD4 cell counts, with rates as high as 17.9 x 100 persons/year and counts of <200 cells/μl [29], although it has also been observed that this can occur with normal CD4 counts [30]. We did not document an association between bacterial community-acquired pneumonia and a history of HAART or with CD4 cell counts, likely due to the inherent limitations of our case series design. In epidemiologic studies performed in the United States and Europe, a decrease in community-acquired bacterial pneumonia rates has been observed after the introduction of HAART. In one study, it was noted that for each month of receiving HAART, the risk for community-acquired bacterial pneumonia decreases by 10% in patients not receiving prophylaxis with TMP/SMX [29]; furthermore, in another study, a decrease of 22.7 to 9.1 cases per 100,000 persons/year was documented following the introduction of HAART in 1997 [31]. A longer time to clinical stability has been associated with advanced HIV infection and with *S*. *pneumoniae* etiology, while receipt of antiretroviral therapy was protective [32]. We did not analyze mortality according to etiologic agents since we were able to isolate pathogens only in 40% of bacterial community-acquired pneumonia. This frequency of pathogen isolation agrees with a recent review [33].

In agreement with previous studies, our results show that in addition to HAART and bacterial community acquired pneumonia, older age, lack of access to social security and CD4 cell counts below 200/mm^3^ were independently associated with all-cause in-hospital mortality. Low CD4 cell counts have been described as associated to a greater risk of mortality probably indicating barriers to health care and to timely diagnosis and treatment initiation [34]. Individuals older than 50 years have been found to be at higher risk of death compared with those aged 19–29 years adjusting by HAART and other epidemiologic and clinical variables [35].

There is evidence that, along with the introduction of HAART, the frequency of AIDS-defining cancer cases has decreased and, concurrently, non-AIDS-defining cancer types, such as respiratory cancer, have increased [36], this is particularly true in developed countries with elevated HAART coverage [37]. However, in Latin American and the Caribbean, cases of AIDS-defining cancer still prevail in HIV infected and AIDS patients, as was observed in the present study [38].

Our study has several limitations, inherent to retrospective studies. Additionally, we were unable to determine time since HIV diagnosis in approximately a third of patients since they were not diagnosed until admission to the hospital. We conducted our study in a specialty hospital for pulmonary diseases. Most of our patient population was referred because of respiratory symptoms from a primary care center that provides care to HIV infected patients in Mexico City. Therefore, our results may not be generalizable to HIV infected patients harboring respiratory diseases attending primary care centers or general hospitals. Also, our patients were mostly young men from low socioeconomic level and therefore our results are not generalizable to women, older individuals or patients from higher socioeconomic strata. Given the median age of participants, this cohort does not seem at risk for more chronic diseases such as chronic obstructive pulmonary disease or lung cancer. Therefore we excluded from analyses non-infectious respiratory illnesses.

The strengths of this study included 1) diagnosis of respiratory diseases according to standardized guidelines; 2) we were able to adjust for important confounding variables and 3) characteristics of our patients most likely reflect limitations of health care access among HIV infected and AIDS patients in similar settings.

### Conclusions

Infectious respiratory diseases still constitute a significant problem for HIV infected and AIDS patients. HAART administered for 180 days or more was associated with a 78% decrease of all-cause hospital mortality. Bacterial community-acquired pneumonia was independently associated with mortality. Almost a third of our study population arrived with advanced disease without having been HIV tested before hospitalization. HIV diagnosis and treatment resources should be improved, and strengthening of HAART program needs to be promoted.

## Supporting Information

S1 Table Type and frequency of discharge respiratory diagnosis among HIV/AIDS patients in a specialty hospital for respiratory diseases in Mexico City, from January 2010 to December 2011. (DOCX)

## References

[1] Mocroft A, Ledergerber B, Katlama C, Kirk O, Reiss P, d'Arminio Monforte A, et al Decline in the AIDS and death rates in the EuroSIDA study: an observational study. Lancet. 2003;362(9377):22–9. Epub 2003/07/11. . 12853195 10.1016/s0140-6736(03)13802-0

[2] Stringer JS, Zulu I, Levy J, Stringer EM, Mwango A, Chi BH, et al Rapid scale-up of antiretroviral therapy at primary care sites in Zambia: feasibility and early outcomes. JAMA: the journal of the American Medical Association. 2006;296(7):782–93. Epub 2006/08/15. 10.1001/jama.296.7.782 . 16905784

[3] Weidle PJ, Malamba S, Mwebaze R, Sozi C, Rukundo G, Downing R, et al Assessment of a pilot antiretroviral drug therapy programme in Uganda: patients' response, survival, and drug resistance. Lancet. 2002;360(9326):34–40. Epub 2002/07/13. 10.1016/S0140-6736(02)09330-3 . 12114039

[4] World Health Organization.Towards universal access: scaling up priority HIV/AIDS interventions in the health sector: progress report, April 2007. Available: http://www.who.int/hiv/mediacentre/universal_access_progress_report_en.pdf. Accessed July, 2015.

[5] Hull MW, Phillips P, Montaner JS. Changing global epidemiology of pulmonary manifestations of HIV/AIDS. Chest. 2008;134(6):1287–98. Epub 2008/12/09. 10.1378/chest.08-0364 . 19059959

[6] Crothers K, Butt AA, Gibert CL, Rodriguez-Barradas MC, Crystal S, Justice AC, et al Increased COPD among HIV-positive compared to HIV-negative veterans. Chest. 2006;130(5):1326–33. Epub 2006/11/14. 10.1378/chest.130.5.1326 . 17099007

[7] Bower M, Powles T, Nelson M, Shah P, Cox S, Mandelia S, et al HIV-related lung cancer in the era of highly active antiretroviral therapy. Aids. 2003;17(3):371–5. Epub 2003/01/31. 10.1097/01.aids.0000050788.28043.cf . 12556691

[8] CENSIDA/Secretaría de Salud 2012, El VIH/SIDA en México 2012. Available: http://www.censida.salud.gob.mx/descargas/biblioteca/VIHSIDA_MEX2012.pdf. Accessed March 2015.

[9] Crabtree-Ramirez B, Caro-Vega Y, Shepherd BE, Wehbe F, Cesar C, Cortes C, et al Cross-sectional analysis of late HAART initiation in Latin America and the Caribbean: late testers and late presenters. PloS one. 2011;6(5):e20272 Epub 2011/06/04. 10.1371/journal.pone.0020272 21637802 PMC3102699

[10] DOF. Norma Oficial Mexicana NOM-168-SSA1-1998, del Expediente Clínico. Available: http://www.salud.gob.mx/unidades/cdi/nom/168ssa18.html. Accessed September, 2014.

[11] Báez SR, Gómez ZC, López EC, et al Neumonía Adquirida en la Comunidad. Revisión y Actualización con una Perspectiva Orientada a la Calidad de la Atención Médica. Neumol Cir Torax 2013; 72- Supl. 1:6–43.

[12] Kaplan JE, Benson C, Holmes KK, Brooks JT, Pau A, Masur H, et al Guidelines for prevention and treatment of opportunistic infections in HIV-infected adults and adolescents: recommendations from CDC, the National Institutes of Health, and the HIV Medicine Association of the Infectious Diseases Society of America. MMWR Recommendations and reports: Morbidity and mortality weekly report Recommendations and reports / Centers for Disease Control. 2009;58(RR-4):1–207; quiz CE1-4. Epub 2009/04/10. . 19357635

[13] Mandell LA, Wunderink RG, Anzueto A, Bartlett JG, Campbell GD, Dean NC, et al Infectious Diseases Society of America/American Thoracic Society consensus guidelines on the management of community-acquired pneumonia in adults. Clinical infectious diseases: an official publication of the Infectious Diseases Society of America. 2007;44 Suppl 2:S27–72. Epub 2007/02/06. 10.1086/511159 . 17278083 PMC7107997

[14] World Health Organization. Guidance on provider-initiated HIV testing and counselling in health facilities. 2007. Available: http://www.who.int/hiv/pub/guidelines/9789241595568_en.pdf. Accessed July, 2015.

[15] DOF. Norma Oficial Mexicana NOM-010-SSA2-2010, Para la prevención y el control de la infección por Virus de la Inmunodeficiencia Humana. Secretaria de Salud, Diario Oficial de la Federación, Mexico, 2010.

[16] Iglesias Chiesa M, Reyes Terán G, Moheno R, Ortiz Monasterio M, Cordero C, Solis E. Evolución del TAR: Mejores fármacos y menos tomas In: Centro de Investigación en Enfermedades Infecciosas, Instituto Nacional de Enfermedades Respiratorias, Fundación México Vivo, Grupo Medios, editors. 30 años del VIH-SIDA Perspectivas desde México. 12011. p. 61–70.

[17] Secretaria de Salud. Guia de manejo antirretroviral de las personas con VIH. Available: http://www.censida.salud.gob.mx/descargas/principal/Guia_ARV_2014V7.pdf. Accessed September, 2014.

[18] Bartlett J, Cheever L, Johnson M, Paauw D. A guide to primary care of people with HIV/AIDS. Department of Health and Human Services, Health Resources and Services Administration, HIV/AIDS Bureau 2004; (1): p. 13–9.

[19] World Health Organization. International Statistical Classification of Diseases and Related Health Problems 10th Revision. Available: http://apps.who.int/classifications/icd10/browse/2015/en. Accessed March 2014.

[20] Tuboi SH, Schechter M, McGowan CC, Cesar C, Krolewiecki A, Cahn P, et al Mortality during the first year of potent antiretroviral therapy in HIV-1-infected patients in 7 sites throughout Latin America and the Caribbean. Journal of acquired immune deficiency syndromes. 2009;51(5):615–23. Epub 2009/05/12. 10.1097/QAI.0b013e3181a44f0a 19430306 PMC2780368

[21] Crothers K, Huang L, Goulet JL, Goetz MB, Brown ST, Rodriguez-Barradas MC, et al HIV infection and risk for incident pulmonary diseases in the combination antiretroviral therapy era. American journal of respiratory and critical care medicine. 2011;183(3):388–95. Epub 2010/09/21. 10.1164/rccm.201006-0836OC 20851926 PMC3266024

[22] Morris A, Lundgren JD, Masur H, Walzer PD, Hanson DL, Frederick T, et al Current epidemiology of Pneumocystis pneumonia. Emerging infectious diseases. 2004;10(10):1713–20. Epub 2004/10/27. 10.3201/eid1010.030985 15504255 PMC3323247

[23] Fischl MA, Dickinson GM, La Voie L. Safety and efficacy of sulfamethoxazole and trimethoprim chemoprophylaxis for Pneumocystis carinii pneumonia in AIDS. JAMA: the journal of the American Medical Association. 1988;259(8):1185–9. Epub 1988/02/26. . 3257532 10.1001/jama.259.8.1185

[24] Palella FJ Jr., Baker RK, Moorman AC, Chmiel JS, Wood KC, Brooks JT, et al Mortality in the highly active antiretroviral therapy era: changing causes of death and disease in the HIV outpatient study. Journal of acquired immune deficiency syndromes. 2006;43(1):27–34. Epub 2006/08/01. 10.1097/01.qai.0000233310.90484.16 . 16878047

[25] Palella FJ Jr., Deloria-Knoll M, Chmiel JS, Moorman AC, Wood KC, Greenberg AE, et al Survival benefit of initiating antiretroviral therapy in HIV-infected persons in different CD4+ cell strata. Annals of internal medicine. 2003;138(8):620–6. Epub 2003/04/16. . 12693883 10.7326/0003-4819-138-8-200304150-00007

[26] Saadani Hassani A, Marston BJ, Kaplan JE. Assessment of the impact of cotrimoxazole prophylaxis on key outcomes among HIV-infected adults in low- and middle-income countries: a systematic review. Journal of acquired immune deficiency syndromes. 2015;68 Suppl 3:S257–69. Epub 2015/03/15. 10.1097/QAI.0000000000000486 . 25768865 PMC4818758

[27] Ledergerber B, Egger M, Erard V, Weber R, Hirschel B, Furrer H, et al AIDS-related opportunistic illnesses occurring after initiation of potent antiretroviral therapy: the Swiss HIV Cohort Study. JAMA: the journal of the American Medical Association. 1999;282(23):2220–6. Epub 1999/12/22. . 10605973 10.1001/jama.282.23.2220

[28] Shorr AF, Susla GM, O'Grady NP. Pulmonary infiltrates in the non-HIV-infected immunocompromised patient: etiologies, diagnostic strategies, and outcomes. Chest. 2004;125(1):260–71. Epub 2004/01/14. . 14718449 10.1378/chest.125.1.260

[29] Kohli R, Lo Y, Homel P, Flanigan TP, Gardner LI, Howard AA, et al Bacterial pneumonia, HIV therapy, and disease progression among HIV-infected women in the HIV epidemiologic research (HER) study. Clinical infectious diseases: an official publication of the Infectious Diseases Society of America. 2006;43(1):90–8. Epub 2006/06/08. 10.1086/504871 . 16758423

[30] Corbett EL, Churchyard GJ, Charalambos S, Samb B, Moloi V, Clayton TC, et al Morbidity and mortality in South African gold miners: impact of untreated disease due to human immunodeficiency virus. Clinical infectious diseases: an official publication of the Infectious Diseases Society of America. 2002;34(9):1251–8. Epub 2002/04/10. 10.1086/339540 . 11941552

[31] Sullivan JH, Moore RD, Keruly JC, Chaisson RE. Effect of antiretroviral therapy on the incidence of bacterial pneumonia in patients with advanced HIV infection. American journal of respiratory and critical care medicine. 2000;162(1):64–7. Epub 2000/07/21. 10.1164/ajrccm.162.1.9904101 . 10903221

[32] Madeddu G, Porqueddu EM, Cambosu F, Saba F, Fois AG, Pirina P, et al Bacterial community acquired pneumonia in HIV-infected inpatients in the highly active antiretroviral therapy era. Infection. 2008;36(3):231–6. Epub 2008/05/09. 10.1007/s15010-007-7162-0 . 18463787

[33] Madeddu G, Laura Fiori M, Stella Mura M. Bacterial community-acquired pneumonia in HIV-infected patients. Current opinion in pulmonary medicine. 2010;16(3):201–7. Epub 2010/02/16. 10.1097/MCP.0b013e3283375825 . 20154625

[34] Lawn SD, Myer L, Harling G, Orrell C, Bekker LG, Wood R. Determinants of mortality and nondeath losses from an antiretroviral treatment service in South Africa: implications for program evaluation. Clinical infectious diseases: an official publication of the Infectious Diseases Society of America. 2006;43(6):770–6. Epub 2006/08/17. 10.1086/507095 . 16912954

[35] Jiang H, Xie N, Cao B, Tan L, Fan Y, Zhang F, et al Determinants of progression to AIDS and death following HIV diagnosis: a retrospective cohort study in Wuhan, China. PloS one. 2013;8(12):e83078 Epub 2014/01/01. 10.1371/journal.pone.0083078 24376638 PMC3871665

[36] Adebamowo CA, Casper C, Bhatia K, Mbulaiteye SM, Sasco AJ, Phipps W, et al Challenges in the detection, prevention, and treatment of HIV-associated malignancies in low- and middle-income countries in Africa. Journal of acquired immune deficiency syndromes. 2014;67 Suppl 1:S17–26. Epub 2014/08/15. 10.1097/QAI.0000000000000255 25117957 PMC4392880

[37] Shiels MS, Pfeiffer RM, Gail MH, Hall HI, Li J, Chaturvedi AK, et al Cancer burden in the HIV-infected population in the United States. Journal of the National Cancer Institute. 2011;103(9):753–62. Epub 2011/04/13. 10.1093/jnci/djr076 21483021 PMC3086877

[38] Fink VI, Shepherd BE, Cesar C, Krolewiecki A, Wehbe F, Cortes CP, et al Cancer in HIV-infected persons from the Caribbean, Central and South America. Journal of acquired immune deficiency syndromes. 2011;56(5):467–73. Epub 2011/01/18. 10.1097/QAI.0b013e31820bb1c3 21239992 PMC3293455

