# Supplementary material for: Association between Highly Active Antiretroviral Therapy and Type of Infectious Respiratory Disease and All-Cause In-Hospital Mortality in Patients with HIV/AIDS: A Case Series
Source: PLoS One. 2015 Sep 17;10(9):e0138115. doi: 10.1371/journal.pone.0138115 (PMC4574922; doi:10.1371/journal.pone.0138115)
Supplement: S1 Table — (DOCX) [file pone.0138115.s001.docx]

**Table S1.** Type and frequency of discharge respiratory diagnosis among HIV/AIDS patients in a specialty hospital for respiratory diseases in Mexico City, from January 2010 to December 2011.

| **Respiratory diagnosis** | **Total population**  **n = 322** |
| --- | --- |
|  | n (%) |
| ***Pneumocystis jirovecii* pneumonia** | **142 (44.1)** |
|  |  |
| **Tuberculosis** | **63 (19.6)** |
| Pulmonary tuberculosis | 41 (12.7) |
| Disseminated tuberculosis | 22 (6.8) |
|  |  |
| **Community-acquired bacterial pneumonia*** | **60 (18.6)** |
|  |  |
| **Mixed** | **23 (7.1)** |
| PJP and pulmonary tuberculosis | 7 (2.2) |
| PJP and cytomegalovirus | 5 (1.6) |
| PJP and aspergillosis | 2 (0.6) |
| Pulmonary tuberculosis and disseminated cryptococcosis | 1 (0.6) |
| Bacterial pneumonia and Kaposi’s Sarcoma | 1 (0.3) |
| Pulmonary tuberculosis and cytomegalovirus | 1 (0.3) |
| Pulmonary tuberculosis and histoplasmosis | 1 (0.3) |
| PJP and disseminated tuberculosis | 1 ( 0.3) |
| Disseminated tuberculosis and cryptococcosis | 1 ( 0.3) |
| Disseminated tuberculosis and Kaposi’s Sarcoma | 1 (0.3) |
| PJP and influenza pneumonia | 1 (0.3) |
| Histoplasmosis and cryptococcosis and MAC pneumonia | 1 (0.3) |
|  |  |
| **Other infectious respiratory diseases** | **20 (6.2)** |
| Influenza virus pneumonia | 5 (1.6) |
| MAC pneumonia | 5 (1.6) |
| Histoplasmosis pneumonia | 4 (1.2) |
| Cryptococcosis pneumonia | 4 (1.2) |
| Cytomegalovirus pneumonia | 1 (0.3) |
| Actinomycosis pneumonia | 1 (0.3) |
|  |  |
| **Non-infectious respiratory diseases** | **14 (4.4)** |
| Kaposi’s Sarcoma | 7 (2.2) |
| Lymphomas | 4 (1.2) |
| Asthma | 2 (0.6) |
| AIDS-non-defining neoplasms (Lung cancer) | 1 (0.3) |

* Includes 6 cases of empyema associated to bacterial pneumonia.
